# Antimicrobial Prophylaxis in Neonates and Children Undergoing Dental, Maxillo-Facial or Ear-Nose-Throat (ENT) Surgery: A RAND/UCLA Appropriateness Method Consensus Study

**DOI:** 10.3390/antibiotics11030382

**Published:** 2022-03-13

**Authors:** Erika Rigotti, Sonia Bianchini, Laura Nicoletti, Sara Monaco, Elena Carrara, Francesca Opri, Roberta Opri, Caterina Caminiti, Daniele Donà, Mario Giuffré, Alessandro Inserra, Laura Lancella, Alessandro Mugelli, Giorgio Piacentini, Nicola Principi, Simonetta Tesoro, Elisabetta Venturini, Annamaria Staiano, Alberto Villani, Enrico Sesenna, Claudio Vicini, Susanna Esposito, on behalf of the Peri-Operative Prophylaxis in Neonatal and Paediatric Age (POP-NeoPed) Study Group

**Affiliations:** 1 Pediatric Unit, Department of Surgical Sciences, Dentistry, Gynecology and Pediatrics, University of Verona, 37124 Verona, Italy; erika.rigotti@aovr.veneto.it (E.R.); opri.francesca@gmail.com (F.O.); roberta.opri@gmail.com (R.O.); giorgio.piacentini@univr.it (G.P.); 2 Pediatric Clinic, University Hospital, Department of Medicine and Surgery, University of Parma, 43126 Parma, Italy; bianchini.sonia@outlook.it (S.B.); laura.nicoletti@studenti.unipr.it (L.N.); s.monaco1410@gmail.com (S.M.); 3 Infectious Diseases Section, Department of Diagnostics and Public Health, University of Verona, 37134 Verona, Italy; elena.carrara@univr.it; 4 Research and Innovation Unit, University Hospital of Parma, 43126 Parma, Italy; ccaminiti@ao.pr.it; 5 Division of Paediatric Infectious Diseases, Department for Woman and Child Health, University of Padua, 35100 Padua, Italy; daniele.dona@unipd.it; 6 Department of Health Promotion, Mother and Child Care, Internal Medicine and Medical Specialties “G. D’Alessandro”, University of Palermo, 90134 Palermo, Italy; mario.giuffre@unipa.it; 7 General Surgery Department, Bambino Gesu Children’s Hospital, Istituto di Ricerca e Cura a Carattere Scientifico (IRCCS), 00165 Rome, Italy; alessandro.inserra@opbg.net; 8 Paediatric and Infectious Disease Unit, Academic Department of Pediatrics, IRCCS Bambino Gesù Children’s Hospital, 00165 Rome, Italy; laura.lancella@opbg.net (L.L.); alberto.villani@opbg.net (A.V.); 9 Department of Neurosciences, Psychology, Drug Research and Child Health, Section of Pharmacology and Toxicology, University of Florence, Viale G. Pieraccini, 6, 50139 Florence, Italy; alessandro.mugelli@unifi.it; 10 Università degli Studi di Milano, 20122 Milan, Italy; nicola.principi@unimi.it; 11 Division of Anesthesia, Analgesia, and Intensive Care, Department of Surgical and Biomedical Sciences, University of Perugia, 06129 Perugia, Italy; simonettatesoro@gmail.com; 12 Pediatric Infectious Disease Unit, Meyer Children’s Hospital, 50139 Florence, Italy; elisabetta.venturini@meyer.it; 13 Department of Translational Medical Science, Section of Pediatrics, University of Naples “Federico II”, 80138 Naples, Italy; staiano@unina.it; 14 Maxillo-Facial Surgery Unit, Head and Neck Department, University Hospital of Parma, 43126 Parma, Italy; enrico.sesenna@unipr.it; 15 Head-Neck and Oral Surgery Unit, Department of Head-Neck Surgery, Otolaryngology, Morgagni Piertoni Hospital, 47121 Forli, Italy; claudio@claudiovicini.com

**Keywords:** dental surgery, ENT surgery, head and neck surgery, maxilla-facial surgery, surgical antimicrobial prophylaxis

## Abstract

Surgical site infections (SSIs) represent a potential complication in surgical procedures, mainly because clean/contaminated surgery involves organs that are normally colonized by bacteria. Dental, maxillo-facial and ear-nose-throat (ENT) surgeries are among those that carry a risk of SSIs because the mouth and the first respiratory tracts are normally colonized by a bacterial flora. The aim of this consensus document was to provide clinicians with recommendations on surgical antimicrobial prophylaxis in neonates (<28 days of chronological age) and pediatric patients (within the age range of 29 days–18 years) undergoing dental, maxillo-facial or ENT surgical procedures. These included: (1) dental surgery; (2) maxilla-facial surgery following trauma with fracture; (3) temporo-mandibular surgery; (4) cleft palate and cleft lip repair; (5) ear surgery; (6) endoscopic paranasal cavity surgery and septoplasty; (7) clean head and neck surgery; (8) clean/contaminated head and neck surgery and (9) tonsillectomy and adenoidectomy. Due to the lack of pediatric data for the majority of dental, maxillo-facial and ENT surgeries and the fact that the recommendations for adults are currently used, there is a need for ad hoc studies to be rapidly planned for the most deficient areas. This seems even more urgent for interventions such as those involving the first airways since the different composition of the respiratory microbiota in children compared to adults implies the possibility that surgical antibiotic prophylaxis schemes that are ideal for adults may not be equally effective in children.

## 1. Introduction

Surgical site infections (SSIs) represent a potential complication in any type of surgical procedure, being associated with prolonged hospital stays and increased postoperative mortality rates, and consequently have a significant medical, social and economic impact [1]. This explains why many if not all surgical procedures have been associated with the administration of antibiotics potentially effective against bacteria that could be responsible for SSIs. In fact, the purpose of reducing the risks of developing SSIs for many years far outweighed the consideration of how effective prophylaxis actually was in various surgical situations and the risks associated with antibiotic use [2]. Hence, the wide use of surgical antibiotic prophylaxis (SAP) in conditions in which this is unnecessary, the use of drugs not suitable for the bacteria potentially present and the prolongation of prophylaxis for much longer than necessary contribute to the emergence of bacterial resistance and excessive health care costs. Moreover, the abuse and misuse of antibiotics for SAP could have consequences that in some cases are more severe than the risk of infection and the emergence of major medical problems which might also prolong hospital stays. The onsets of acute renal failure and antibiotic-associated diarrhoea (i.e., *Clostridium difficile* colitis) remain some of the most significant examples in this regard [3]. The awareness of these problems has led many experts to revise the methods of administrating SAP and to draw up guidelines to rationalize its use in each type of surgery. However, at present, not all aspects of the problem have been precisely defined. For many types of surgery, there is a lack of data derived from controlled clinical trials necessary to define with certainty whether and how to perform SAP. This explains why medical behaviors remain very heterogeneous and often different from what is suggested by official recommendations [4,5].

Many of the recommendations that can be debated concern surgery that is considered clean/contaminated because it involves organs that are normally colonized by bacteria. Dental, maxillo-facial and ear-nose-throat (ENT) surgeries are among these because the mouth and the first respiratory tracts are normally colonized by a rich bacterial flora, including potential pathogens that can result in SSIs [6,7]. The known limitations are even more evident for children. Pediatric studies of SAP are extremely hard to come by. Moreover, the respiratory microbiota in children is different from that of adults and susceptibility to respiratory infection varies significantly in different pediatric ages [8]. Therefore, the aim of this consensus document was to provide clinicians with recommendations on SAP in neonates (<28 days of chronological age) and pediatric patients (within the age range of 29 days–18 years) undergoing dental, maxillo-facial or ENT surgical procedures. This consensus may lead to two advantages: to clarify if and how to perform SAP in these subjects and to indicate which fields deserve significant in-depth studies and new research.

## 2. Methods

### 2.1. RAND/UCLA Appropriateness Method

The consensus document was realized using the Research and Development Corporation (RAND) and the University of California—Los Angeles (UCLA) appropriateness method. The RAND/UCLA method consists of the evaluation of the appropriateness of diagnostic and therapeutic procedures with sub-optimal scientific evidence by a panel of experts [9]. According to the RAND method, a procedure is defined as “appropriate” if the expected benefits outweigh the expected negative consequences, with a wide margin that justifies it, regardless of the costs. On the contrary, a procedure whose expected risks outweigh the expected benefits is considered as “inappropriate”. According to the RAND definition, the expert who makes an appropriateness/inappropriateness judgment must consider the clinical benefits and not be influenced by economic considerations. Therefore, the appropriateness accounts for the evaluation of the risk/benefit ratio of a list of management and therapeutic procedures [10]. For a heterogeneous topic such as surgical antimicrobial prophylaxis on which randomized controlled trials in pediatrics are lacking, the application of methods aiming to increase the homogeneity of behaviors by neonatologists, infectious diseases specialists, pediatric surgeons and anesthetists appeared useful and appropriate. For this reason, the RAND/UCLA approach was chosen instead of the GRADE methodology. Through the RAND method, the participants discussed different clinical scenarios and elaborated statements on the basis of the literature and their clinical experience. The group of experts did not consider it appropriate to combine the GRADE method with the RAND/UCLA approach because the absence of randomized studies represents a bias in defining the strength of the recommendations and in representing a consensus reached for real-life situations.

### 2.2. Recruitment of the Expert Panel

A multidisciplinary group of experts belonging to the main Italian scientific societies dealing with anti-infective therapy in pediatric ages was selected. The following scientific societies were involved: the Italian Society of Pediatrics (SIP), the Italian Society of Neonatology (SIN), the Italian Society of Pediatric Infectious Diseases (SITIP), the Italian Society of Infectious and Tropical Diseases (SIMIT), the Italian Society of Pediatric Surgery (SICP), the Italian Society of Microbiology (SIM), the Italian Society of Pharmacology (SIF), the Italian Society of Anesthesia and Neonatal and Pediatric Resuscitation (SARNEPI) and the Italian Society of Childhood Respiratory Diseases (SIMRI). The panel of experts was made up of 52 medical doctors with at least 5-years of experience: pediatricians (n = 20), neonatologists (n = 6), infectious diseases specialists (n = 5), pediatric surgeons (n = 3), a maxillo-facial surgeon (n = 1), an otolaryngologist surgeon (n = 1), anesthetists (n = 8), pharmacologists (n = 5) and microbiologists (n = 3).

### 2.3. Generation of Scenarios

Initially, a literature search was performed with the selection of documents including randomized studies, systematic reviews of the literature, meta-analyses and guidelines on peri-operative prophylaxis for the prevention of SSI in neonatal and pediatric dental, maxillo-facial or ENT surgery. The literature search was carried out on the PubMed Database, with only articles published in English from the year 2000 to 2020 being chosen. The following key terms were used: “antimicrobial prophylaxis” OR “antibiotic prophylaxis” AND “dental surgery” OR “dental” OR “teeth” OR “gum tissue” OR “oral mucosa” OR “maxillo-facial” OR “mandible” OR “maxillary” or “zygomatic” OR “temporo-manidibular” OR “cleft palate” OR “cleft lip” OR “ear surgery” OR “tympanostomy” OR “tympanoplasty” OR “stapedectomy” OR “ear tube placement” OR “cochlear implant” “paranasal surgery” OR “rhinosinus surgery” OR “head surgery” OR “neck surgery” OR “thyroidectomy” OR “parathyroidectomy” OR “salivary gland surgery” OR “parotidectomy” OR “submandibular gland excision” OR “laryngectomy” OR “pharyngectomy” OR “tracheotomy” OR “neck dissection” OR “lymphangiomas exeresis” OR “neck cysts excision” or “fistulas excision” OR “laser airway surgery” OR “tonsillectomy” OR “adenoidectomy” OR “adenotonsillectomy” OR “septoplasty” AND “neonate” OR “newborn” OR “paediatric” OR “pediatric” OR “children” OR “adolescent”. Subsequently, using the Patient/Problem/Population-Intervention-Comparison/Control/Comparator-Outcome (PICO) model (i.e., defining a clinical question in terms of the specific patient problem), a questionnaire was created on SAP in neonatal and pediatric dental, maxillo-facial and ENT surgery, and was divided into nine clinical scenarios. All neonatal and pediatric dental, maxillo-facial and ENT surgical procedures were considered. Before administration, the questionnaire was tested twice with a one-week interval to a convenience sample of four pediatricians, two neonatologists, one infectious diseases specialist, one pediatric surgeon, one maxillo-facial surgeon, one otolaryngologist surgeon, one anesthetist, one pharmacologist and one microbiologist. Then, 26 out of 52 experts were selected by the scientific societies and the questionnaire was administered to 11 pediatricians, 3 neonatologists, 2 infectious diseases specialists, 1 pediatric surgeon, 1 maxillo-facial surgeon, 1 otolaryngologist surgeon, 4 anesthetists, 2 pharmacologists and 1 microbiologist.

### 2.4. Two-Round Consensus Process

On the basis of the scenarios, the questionnaire was submitted to the experts on the “REDCap” online platform. Each question included the clinical scenario and possible answers relating to whether or not SAP was recommended for the scenario, and, in case of its recommendation, a list of all the antibiotics available on the EU market was included so that the expert could select the antibiotics that he/she considered as their first choice. The selected bibliographic material was made available to all panel members, who were instructed on how to fill in the panel. The experts answered the questionnaire anonymously and their judgments were expressed on a 1–9 scale, where “1” was considered definitely inappropriate, “5” was considered uncertain and 9 was considered definitely appropriate. Intermediate values corresponded to different modulations in the judgment in terms of inappropriateness (“2” and “3”), uncertainty (from “4” to “6”) and appropriateness (“7” and “8”), respectively. When evaluating each indication, each expert could refer to both his/her own experience and clinical judgment and the available scientific evidence. A free space was provided for any annotations or comments.

The first round of the questionnaire was blind to other panel members. The results of the survey were discussed in a collegial meeting in order to find agreements and reduce eventual disagreements. Clarifications, adaptations and refinements of the indications and appropriateness ratings were made. A total of nine recommendations were developed.

Participants were asked to approve the recommendations in a second round during the following four weeks. During round two, the level of consensus within the panel for each scale for each scenario was calculated in real-time. Mean values and disagreements were classified in terms of three levels of appropriateness (appropriate: between ‘7’ and ‘9’, without disagreement; uncertain: between ‘4’ and ‘6’ or any median with disagreement; inappropriate: between ‘1’ and ‘3’, with agreement). Agreement was reached when at least 75% of participants ranked within the same level of appropriateness.

## 3. Results

### 3.1. SCENARIO #1. Dental Surgery

For years, it was believed that dental surgery, in addition to carrying the risk of local infections at the site of surgery, could lead to bacteremia and, consequently, could promote the development of distant infections [11]. The pathogens responsible for these problems were identified mainly in viridans streptococcal species, followed by *Staphylococcus aureus* and *Enterococcus* spp. [12]. Over time, it has been shown that the risk of bacteremia is very low, can be taken into account only for subjects with cardiological or orthopedic problems and can be considered marginal in all other cases. The acquisition of new information has modified the recommendations of scientific societies on SAP for subjects undergoing dental surgery, becoming increasingly restrictive.

With regard to the prevention of bacterial endocarditis in subjects with heart disease, it is now accepted by most scientific societies that the risk of developing endocarditis following dental surgery is lower than that which occurs when brushing teeth, chewing gum for hours or using toothpicks [13,14]. Moreover, it seems to be well established that the benefits of administering antibiotics prior to dental surgery are minimal or even non-existent and may, in any case, not justify the harm associated with the use of these drugs [13,14]. A study conducted in Taiwan that monitored the development of bacterial endocarditis in the entire population over 10 years and attempted to correlate it with the performance of dental surgery was unable to demonstrate any relationship between the two variables [15]. Hence, the authors recommended that the proper and continuous cleaning of the mouth and teeth was maintained as a basic element to reduce the risk of bacterial endocarditis, thus avoiding any form of SAP in dental surgery.

However, uncertainties remain with regard to a selected group of patients undergoing specific forms of dental surgery. The American Heart Association [16] and the American Academy of Pediatric Dentistry [17] suggest that SAP may be considered when the surgery involves the manipulation of the gum tissue or periapical region of the teeth or involves the perforation of the oral mucosa. This is indicated, in particular, if the subject has already suffered from endocarditis, has already been operated on with the application of prosthetic material, has a cyanogenic congenital heart disease that has not yet completely repaired, has a congenital heart disease that has already been repaired with the application of prosthetic material in the 6 months following surgery and has been transplanted and has developed valvulopathy. When necessary, it is recommended that SAP should be conducted prior to the performance of surgery without further continuation of antibiotic administration. Amoxicillin per os, ampicillin or cefazolin ev or, in subjects with a penicillin allergy, cephalexin per os or clindamycin ev are considered the drugs of choice [13].

The problem of SAP is equally or even more controversial in dental procedures among patients with orthopedic problems, particularly those with joint prostheses. Also, in cases such as these, the increased risk of infection and the usefulness of SAP for the prevention of prosthetic infections have been overestimated for years. In fact, the most recent and best-performed studies seem to deny any relationship between dental surgery and the development of prosthetic infection [18,19,20]. This explains why the most recent guidelines [21,22,23,24] do not recommend SAP for dental procedures in individuals with joint prostheses. Despite this, the majority of dentists and orthopedists continue to use antibiotic prophylaxis, ignoring the recommendations of scientific societies [25,26].

Recommendation 1. In the case of a pediatric patient undergoing dental surgery, no perioperative antibiotic prophylaxis is recommended. Oral amoxicillin or ampicillin ev (50 mg/kg for both) should be administered during the 30 min before surgery if the operation involves the manipulation of gum tissue or the periapical region of the teeth or involves the perforation of the oral mucosa and the subject has already suffered from endocarditis, has already been operated on with the application of prosthetic material, has a cyanogenic congenital heart disease not yet fully repaired, has a congenital heart disease already repaired with the application of prosthetic material in the 6 months following surgery or has been transplanted and has developed valvulopathy. No prophylaxis is recommended in subjects with prosthetic implants.

### 3.2. Maxillo-Facial Surgery

Several forms of surgery fall under the category of maxillo-facial surgery. The usefulness of SAP in these cases can vary considerably depending on the type of surgical procedure [27].

#### 3.2.1. SCENARIO #2. SURGERY Following Trauma with Fracture

A number of studies have shown that the risk of SSIs depends on the type and site of the fracture, with it being greater if the bone fracture is in communication with the oral cavity or skin surface and involves the mandible rather than the maxilla. Studies evaluating the usefulness of SAP have shown that the pre-operative administration of antibiotics may be useful in surgeries involving the mandible, while there are no benefits in those involving the upper and middle portions of the face [28,29]. In any case, post-operative prophylaxis is unnecessary. These conclusions derive from two meta-analyses of studies conducted almost exclusively in adults. In the first one, which analyzed four studies published before 2006, it was shown that the infectious risk is three times lower in subjects undergoing surgery for fracture of the mandible who receive pre-operative SAP in a single dose or continued administration for 24 h compared to those not treated [28]. In contrast, SAP does not result in reduced infectious risk in mandibular condyle, maxilla or zygomatic fracture. Although several antibiotics have been shown to be effective, the one most often used was oral amoxicillin. In the second meta-analysis, which included 13 studies published before 2019 and compared pre-operative prophylaxis or continued administration for 24 h with SAP maintained for several days after surgery, it was shown that the latter did not result in any significant reduction in the risk of SSIs (relative risk [RR]: 1.11, 95% confidence intervals [CI]: 0.86–1.44; *p* > 0.1) [28]. No advantage was demonstrated when the analysis was restricted to the site of surgery or the type of injury (mandibular fracture, RR: 1.22; 95% CI: 0.92–1.62; maxilla fracture RR, 1.02, 95% CI: 0.62–1.67). With pediatric studies lacking, recommendations in children are those followed in adults.

Recommendation 2. In the case of a pediatric patient undergoing maxillo-facial fracture surgery, pre-operative antibiotic prophylaxis with oral amoxicillin 50 mg/kg is recommended within 30 min prior to surgery when the surgery involves the mandible. Prophylaxis is not recommended in case of maxillary or zygomatic surgery.

#### 3.2.2. SCENARIO #3. Temporo-Mandibular Surgery

Arthroplasty, condylectomy and, in recent years in pediatrics, total joint replacement surgery all fall into this group. The risk of SSIs is calculated at 1.5–4.5%, with *S. aureus*, *S. epidermidis* and *Peptostreptococcus* spp. as the main pathogens. Despite lacking reliable demonstrations of the benefits of SAP, some authors suggest the administration of antibiotics active against these bacteria at least pre-operatively [30,31,32]. Intravenous (EV) cefazolin is recommended in these cases.

Orthognathic surgery includes reconstructive surgery, maxillomandibular advancement and surgical correction of facial asymmetry. Corticotomy, osteotomy and the placement of distraction devices for the treatment of Pierre Robin syndrome are the best examples of children [33]. Studies regarding prophylactic antibiotic use in this setting are few and the results are conflicting. Those with the lowest risk of bias seem to indicate that preoperative antibiotics can be effective, whereas postoperative antibiotics have no role to play [34,35].

Recommendation 3. In the case of a pediatric patient undergoing temporo-mandibular surgery, pre-operative antibiotic prophylaxis with cefazolin in a single dose of 30 mg/kg (maximum dose 2 g) EV is recommended in the 30 min before surgery.

#### 3.2.3. SCENARIO #4. Cleft Palate and Cleft Lip Repair

The development of SSIs after cleft lip or cleft palate surgery can cause clinical problems of extreme importance both immediately and in long term. Besides the risk of bacteremia and the infection of distant organs and systems, there is the local risk of the relevant dehiscence of the wound, with serious repercussions on facial aesthetics, speech development and, in case of cleft lip, the creation of palatal fistulas, all conditions that may require further surgery [36]. Nevertheless, few studies have exactly quantified this risk and the possible benefits derived from the use of SAP. Some data have been collected in patients undergoing cleft palate repair, but the results are far from conclusive. A retrospective study conducted by the American Cleft Palate-Craniofacial Association, in which the postoperative course of 311 patients was analyzed, of whom only 173 had received SAP, showed that surgery itself carries a significant risk of delayed wound healing and palatal fistula development and that the administration of SAP does not reduce this risk [37]. The development of delayed wound healing was demonstrated in 16.8% of subjects on SAP and in 15.2% of those without (*p* = 0.71). The appearance of palatal fistulas was observed in 2.9% versus 1.4% of the cases, respectively (*p* = 0.47). Results apparently in favor of SAP were collected in a prospective, randomized, double-blind, placebo-controlled study conducted in India [38]. However, even in this case, in which a slightly lower incidence of SSIs was documented in subjects undergoing short-term and long-term antibiotic prophylaxis (13.8% versus 8.7% for SSIs in short-term and 17.1% versus 10.7% for fistulas in long-term), the differences between the two groups were not statistically significant (*p* = 0.175 and *p* = 0.085, respectively) and the authors’ conclusions were uncertain regarding the efficacy of SAP.

The scarcity of specific studies and the lack of results capable of definitively establishing the importance of SAP explain why there are currently no internationally accepted guidelines indicating the most appropriate approach to cleft lip or cleft palate repair and why, in clinical practice, surgeons in various centers have very different behaviors, even if, in general, they are in favor of SAP. In the study of the American Cleft Palate-Craniofacial Association, it is reported that SAP was not applied in only 15% of operated patients, while the SAP schemes used in 85% of treated subjects and consisted of a single pre-operative dose in 26% of treated patients and multiple administrations in the others [38]. Specifically, antibiotic administration continued for 24 h beyond the end of surgery in 23% of cases, for 25–72 h in 12%, for 4–5 days in 16% and for 6–10 days in 12%. A first-generation cephalosporin was used in 64% of the cases, ampicillin/sulbactam in 13%, clindamycin in 8% and penicillin in 5%. All of this is in contrast to what is expected based on the consideration that cleft lip and cleft palate repair surgeries should be considered clean surgeries and, therefore, should not require antibiotic prophylaxis. On the other hand, the widespread fear of SSI development with dramatic consequences may explain the widespread use of SAP in these cases. Microbiological data show that, in general, SSIs following these surgeries are sustained by the same bacteria present in the oral cavity [36] and that the preoperative presence of *S. pyogenes* and *S. aureus* seems to be a significant risk factor for the development of SSIs [39]. The antibiotics recommended must, therefore, be effective against these bacteria. In this regard, the American Cleft Palate-Craniofacial Association suggests the use of ampicillin/sulbactam. With a lack of convincing data on the use of multiple doses, the administration of a single pre-operative dose is recommended [38].

Recommendation 4. In the case of a neonatal or pediatric patient undergoing cleft lip or cleft palate correction surgery, peri-operative prophylaxis with ampicillin/sulbactam at a dose of 50 mg/kg (as ampicillin) EV is recommended to be administered within 30 min before surgery.

### 3.3. ENT Surgery

#### 3.3.1. SCENARIO #5. Ear Surgery

There are numerous surgical procedures that fall within ear surgery. All those that are performed in the absence of ongoing infectious processes, such as those involving the insertion of tympanostomy tubes, tympanoplasty and stapedectomy in subjects without infection of the ear canal and/or middle ear, are among the so-called clean procedures that, by definition, are associated with a low or no risk of SSI development [40]. On the other hand, surgeries performed in subjects with chronic infectious middle ear disease with or without cholesteatoma or involving drainage from an infected site, including transtympanic tube placements, are considered clean/contaminated or simply contaminated surgeries and are generally associated with a high risk of SSI development [40]. Studies that quantified the true magnitude of this risk have shown that in clean surgeries, less than 5% of operated subjects experience SSIs, whereas in clean/contaminated or contaminated surgeries, this value rises to more than 10% [40]. The pathogens involved are, in addition to *S. aureus*, all those commonly involved in the determination of otologic infections, including those that may play a role in chronic suppurative otitis media, such as *Pseudomonas aeruginosa* [41]. Given this, it would be easy to infer whether and what SAP to use in the various forms of ear surgery. However, the available studies, often burdened by important methodological limitations, do not permit any conclusions to be drawn. The major limitation is represented by the fact that in many studies, the evaluation of the usefulness of SAP includes subjects with different pathologies, thus adding cases of clean intervention to cases of contaminated intervention. Moreover, the comparison between SAP limited to a single pre-operative administration and long-term prophylaxis after surgery is often conducted with different drugs and dosages [42,43,44,45,46,47]. A systematic review of the literature available up to the end of 2009, in which the use of SAP in clean and clean/contaminated otologic surgery was evaluated, showed that SAP in these conditions is completely unnecessary [48]. The incidence of SSIs was generally low in all cases, with no differences between those who had received the placebo, those who received peri-operative prophylaxis and those treated with prolonged postoperative prophylaxis [48]. In the absence of firm data, the rationale generally followed in clean versus clean/contaminated or simply contaminated surgeries prevails. In the former, prophylaxis is not recommended; in the latter, the use of pre-operative prophylaxis with cefazolin EV is recommended [49].

The use of SAP in the case of cochlear implant surgery is a separate issue. Initially considered possible in up to 40% of cases, infections secondary to cochlear implantation are now limited to 1.4–8.2% of cases in many patients months after surgery [50]. Improved surgical techniques and materials used for implantation are considered the main reasons for this change. However, although relatively uncommon, infections following cochlear implantation can have dramatic consequences, far greater than those that can result from other forms of ear surgery. Infections can result in the need to remove the device and perform a second surgery [51], and infection can spread to the interior of the skull with the development of meningitis and abscesses [52]. Theoretically, cochlear implant placement is a clean procedure that may not require prophylaxis [53,54,55]. However, the risk of dramatic complications dictates a careful evaluation of the importance of SAP. Unfortunately, there are no randomized clinical trials performed with appropriate methods that can clarify this point. A systematic review of the literature published in this regard identified only three retrospective studies, all burdened by considerable heterogeneity and not negligible methodological limitations [56]. These studies seem to indicate that no form of SAP is useful in modifying the low tendency to develop SSIs in subjects undergoing cochlear implantation, suggesting no use of SAP in these cases [56]. In reality, these data are not convincing, as demonstrated by the fact that some authors believe they should suggest a different choice depending on the characteristics of the individual patient and some prestigious scientific institutions recommend, for caution, a systematic peri-operative prophylaxis with cefazolin EV [57]. Also discussed is how to perform SAP, whether with a single pre-intervention dose or with a more or less protracted antibiotic administration after surgical wound closure. A recent French study showed that a short treatment is ideal for adults, while a protracted one is more effective in children [57]. However, our panel of experts considered pre-operative antibiotic prophylaxis with a single dose of cefazolin EV as more appropriate, as recommended for clean/contaminated or contaminated ear surgery [49].

Recommendation 5. In the case of a neonatal or pediatric patient undergoing ear surgery, peri-operative antibiotic prophylaxis is not recommended for clean surgery, whereas it is recommended in cases of clean/contaminated or contaminated operation and for cochlear implant placement. When antibiotic prophylaxis is indicated, it is recommended to administer cefazolin as a single dose of 30 mg/kg (maximum dose 2 g) EV within 30 min before surgery.

#### 3.3.2. SCENARIO #6. Endoscopic Paranasal Cavity Surgery and Septoplasty

Endoscopic surgery of the rhinosinus cavities by definition falls into clean-contaminated surgeries or even into contaminated ones in cases with bacterial rhinosinusitis [58]. It should, therefore, be among the surgical procedures for which SAP should be provided. Several studies have shown that patients undergoing endoscopic rhinosinus cavity surgery frequently harbor pathogens such as *S. aureus*, anaerobes and *S. pneumoniae* in the examined sinuses [58,59,60,61]. Moreover, the procedure may be followed by bacteremia in a number of cases (7%) [62]. This suggests the possibility of developing sepsis and other significant infections at a distance from the site of surgery with very clinically relevant outcomes. Nevertheless, in the guidelines prepared by the American Society of Health-System Pharmacists, the Infectious Diseases Society of America, the Surgical Infection Society and the Society for Healthcare Epidemiology of America, this form of surgery, along with tonsillectomy, is excluded from the recommendations for SAP [63]. In reality, there are no firm data indicating the reasons for this choice. Studies quantifying the risk of developing SSIs are practically absent. In addition, they do not provide information on pre-operative prophylaxis, considering only the administration of antibiotics for days or weeks after surgery. In three studies, administration of cefuroxime for 10 days [64], amoxicillin/clavulanic acid for 3 weeks [65] or amoxicillin for 4 weeks [66] did not yield results different from those seen in patients who had received a placebo. In contrast, in another study [67], the use of amoxicillin/clavulanic acid for 2 weeks allowed better results in terms of endoscopic findings at both 5 and 12 days. However, the use of SAP in endoscopic surgery is widely practiced by surgeons. A survey conducted among members of the American Rhinologic Society showed that 20.6% routinely performed preoperative prophylaxis, 54.4% intraoperative prophylaxis and 62.3% postoperative prophylaxis [68]. There are, however, no definitive data available on the actual efficacy of SAP in pediatric subjects undergoing endoscopic rhinosinus surgery [69]. Pending specific studies, and given the potential risk of development of infectious complications, our expert panel believed that pre-operative antibiotic prophylaxis with a single dose of cefazolin EV may be recommended in children undergoing rhinosinus endoscopy surgery. Regarding septoplasty, studies demonstrating the necessity or effectiveness of antibiotic prophylaxis are few. However, available data seem to indicate that if septoplasty increases *S. aureus* colonization and reduces normal flora, pre-operative antibiotic administration does not protect against potential pathogen colonization and contributes to a further decrease in normal rhinopharyngeal microbiota [70]. Therefore, SAP is not recommended in septoplasty [71].

Recommendation 6. In the case of a pediatric patient undergoing endoscopic surgery of the rhinosinus cavities, it is recommended to administer peri-operative antibiotic prophylaxis with cefazolin 30 mg/kg (maximum dose 2 g) EV within the 30 min before surgery. No antibiotic prophylaxis is recommended in septoplasty.

### 3.4. Head and Neck Surgery

#### 3.4.1. SCENARIO #7. Head and Neck Clean Interventions 

The majority of head and neck surgeries are considered clean surgeries and are followed by the development of SSIs in less than 1% of cases [72]. These include thyroidectomy, parathyroidectomy, salivary gland surgeries, the removal of lymphangiomas and the excision of lateral and medial neck cysts and fistulas. For these, no SAP is recommended, also because the available studies, all referring to adult patients, seem to indicate that the administration of antibiotics either pre-, intra- or post-operatively does not reduce the already low frequency of the occurrence of SSIs [72,73]. A separate evaluation is suggested by some authors for neck dissection surgery that, although classified as clean surgery, is associated with a slightly higher risk of SSIs because it involves a higher degree of tissue exposure. However, studies aimed at quantifying the true risk of SSIs in this type of surgery [74,75] and those aimed at measuring the impact of perioperative antibiotic prophylaxis [76,77] do not definitively clarify the characteristics of this type of surgery. In fact, the data collected from a small number of studies performed with different (and sometimes contradictory) methods are largely contrasting. The problem remains open, although some authors indicate that in these conditions, the use of pre-operative antibiotic prophylaxis with post-operative prolongation after surgical wound closure for less than 24 h could be recommended [78]. In any case, data specific to pediatric aged patients are lacking. Therefore, the recommendations provided for adults are considered valid also for children and our expert panel agreed to not recommend SAP for this type of surgery.

Recommendation 7. No perioperative antibiotic prophylaxis is recommended in the case of a neonatal or pediatric patient undergoing clean head and neck surgery (i.e., thyroidectomy, parathyroidectomy, salivary gland surgeries, the removal of lymphangiomas and the excision of lateral and medial neck cysts and fistulas).

#### 3.4.2. SCENARIO #8. Head and Neck Clean-Contaminated Interventions

All surgeries on head and neck structures that involve the opening of the airway or gastrointestinal tract (i.e., oral cavity resection, laryngectomy, pharyngectomy, tracheotomy and maxillary of upper airways tumor masses) are considered clean-contaminated [79]. It has been shown that such surgeries are followed by SSIs in 25–85% of cases [80]. The bacteria most often responsible are the same ones that normally colonize the mouth and pharynx, with the highest frequencies found for *Streptococcus* spp. (aerobes and anaerobes), *S. aureus*, *Bacteroides* spp. (with the exception of *B. fragilis*), *Fusobacterium* spp., *Peptostreptococcus* spp. and *Veillonella* spp. [79,80]. In addition, it has been demonstrated that SAP is extremely effective in reducing subsequent infections. A meta-analysis of 12 studies conducted before 1991 had already quantified the reduction in the frequency of SSIs as 43.7% when peri-operative antibiotic prophylaxis was used, with the greatest advantage associated with long-term prophylaxis over single-dose prophylaxis [81]. More recent studies have confirmed the usefulness of peri-operative prophylaxis, although they have not fully clarified which antibiotics and which form of prophylaxis might be the most effective. In particular, peri-operative prophylaxis is recommended for patients undergoing parotid gland surgery, and intravenous antibiotics during the post-operative course are highly suggested in case of patients with a histories of previous acute parotid infection and drain output ≥ 50 mL in the first 24 h [82]. As for the drug(s), a huge number of studies are available. The relative quality of many of them and the low number of subjects enrolled in others make it impossible to indicate which single antibiotic or combination might be recommended. On the other hand, it is not uncommon for similar studies to have yielded conflicting results, with there being a further difficulty in identifying the ideal antibiotic prophylaxis. What seems to be established is that antibiotic prophylaxis should be implemented with drugs or associations that are active on Gram-positive and Gram-negative bacteria and have good coverage against anaerobes. Clindamycin alone or in combination with other compounds active on both Gram-positive and Gram-negative bacteria [83,84,85], cefazolin alone or with metronidazole [86,87,88], other cephalosporins [89,90,91] and the combinations of amoxicillin/clavulanic acid [92,93] and ampicillin/sulbactam [94,95,96] are the most widely tested forms of antibiotic prophylaxis with no clear superiority. With regard to the duration of administration, it is not possible to draw definitive conclusions about the efficacy of a single dose of antibiotics before the start of surgery because studies in this regard are too limited. On the contrary, it seems certain that a prophylaxis that extends beyond the closure of the intervention site is useful, even if it is not clear how long the administration should be prolonged. Indeed, there are no significant differences in prevention implemented with a 24-h or a 3-, 5- or 7-day prolongation [97,98,99]. This has led to recommendations for the use of cefazolin or cefuroxime associated with metronidazole or ampicillin/sulbactam as the first choice to be administered before surgery and immediately afterwards for no more than 24 h. Because all the studies were conducted almost exclusively on adults, recommendations for children can only be derived from that evidence.

Recommendation 8. In the case of a neonatal or pediatric patient undergoing clean-contaminated ENT surgery (i.e., oral cavity resection, laryngectomy, pharyngectomy, tracheotomy or the removal of upper airways tumor masses), peri-operative antibiotic prophylaxis with cefazolin 30 mg/kg (maximum dose 2 g) EV administered within 30 min before surgery combined with metronidazole 15 mg/kg (max 500 mg) is recommended.

### 3.5. SCENARIO #9. Tonsillectomy and Adenoidectomy

For many years since the beginning of the antibiotic era, it was believed that SAP was an essential measure to reduce the risk of post-operative problems, including SSIs, in patients undergoing tonsillectomy. In fact, a survey of US otolaryngologists in 2004 showed that nearly 80% of them prescribed antibiotics to subjects scheduled for tonsillectomy [100]. More recent research and the demonstration that many of the studies that had led to the use of SAP were burdened by severe methodological limitations have completely reversed the initial assessments, leading to completely different recommendations [101,102,103,104,105,106,107,108]. Current knowledge is well summarized by the results of the meta-analysis of 10 randomized controlled trials conducted before 2012, which clearly highlighted that SAP does not reduce postoperative pain, the need for pain medication or the risk of bleeding [101]. In the few studies in which the administration of antibiotics seemed to be somewhat effective, the benefits were extremely modest. Subsequent studies confirmed these results, pointing out that SAP had no advantage even in reducing emergency room admissions or hospitalization [102,103,104,105,106,107,108]. All of this explains why the American Academy of Otolaryngology-Head and Neck Surgery Foundation recently reiterated in its guidelines for tonsillectomy in otherwise healthy children that SAP administration in these subjects should not be used at all [106]. Exceptions may be made for subjects at high risk of the onset of serious infectious problems (i.e., subjects with pre-existing cardiologic pathology already identified as requiring antibiotic prophylaxis in case of surgery).

Overlapping conclusions can be made for adenoidectomy, alone or in association with tonsillectomy. A number of studies have shown that in both of these conditions, surgery can be associated with bacteremia and, therefore, with the potential risk of sepsis or localization of the infection at a distance from the oral cavity [107]. However, while the risk of bacteremia is undeniable, with *Haemophilus influenzae*, viridans streptococcal species, *S. pneumoniae* and *S. aureus* as the most common pathogens [108,109], this seems entirely transient and not remotely followed by the development of major infectious issues. A study comparing subjects undergoing adenoidectomy with and without SAP showed that antibiotics were markedly helpful in reducing the risk of bacteremia at 30 s after the end of surgery (3.9% in treated versus 32.7%; *p* < 0.001), but that this difference was no longer significant in controls performed at 20 min after surgery (3.9% versus 14.3%; *p* = 0.089). In addition, both in the short- and long-term after surgery, the risk of complications of any kind, including acute otitis media, proved to be extremely low in each case and not different in the two groups [110].

Recommendation 9. In the case of a pediatric patient undergoing tonsillectomy, adenoidectomy or both, no antibiotic prophylaxis is recommended.

## 4. Discussion

Many clinical conditions requiring surgical procedures are relatively uncommon in pediatrics. This explains why in these cases, studies on the necessity and efficacy of SAP to reduce SSIs are very few or completely absent and the recommendations for the use of this preventive measure for pediatric aged patients are simply derived from those provided for adults. This seems to be somewhat different in the case of dental, maxillo-facial or ENT surgery because some of the surgical procedures in these areas are extremely common (Table 1). This is the case in cleft palate and cleft lip correction, tonsillectomy, adenoidectomy and transtympanic tube placement. In reality, recommendations are well-defined and shared by all experts in only very few conditions. Generally, studies are few and far between, methodologically questionable and provide different results.

In these cases, SAP is recommended because the intervention could be at risk of serious complications even if it is not clear that it is really necessary. Typical examples in this regard are given by SAP for cleft palate or cleft lip correction surgery and for cochlear implant placement. In these cases, conclusive data are lacking, and the fear of serious complications ends up being the main motivation for the systematic use of antibiotics and very poor adherence to suggested recommendations in everyday surgical practice. More precise and definitive recommendations can only be given for tonsillectomy and adenoidectomy procedures because the in-depth study of the infectious risks associated with these procedures have been extensively defined. In these cases, the recommendation to not perform SAP is precisely supported by the evidence that the bacteremia following surgery is of very short duration and not followed by further localization.

The specific scenarios developed are intended to guide healthcare professionals in practice, so as to ensure the improved and standardized management of neonatal and pediatric patients. The strengths of the work are an updated literature review, the use of a rigorous analysis method (RAND/UCLA), the involvement of a large number of exponents of the most important Italian scientific societies and the specific consideration of neonatal aged patients. The potential limitation of the work is the scarcity of data in the literature, which is partly overcome by the involvement of numerous and selected experts. On the other hand, the lack of pediatric studies on the selected topics did not permit the use of the GRADE methodology and the complexity of the topics required an online one-to-one meeting with all the participants.

Table 2 shows SAP for neonates and children undergoing dental, maxillo-facial or ENT surgeries. Antibiotic dosages are those routinely recommended [111]. Although the neonatal pharmacokinetics differs depending on gestational age, body weight and days after birth, the panel of experts did not recommend changes to the doses because of the short exposure duration of antimicrobial prophylaxis and the safety of the recommended drugs in patients of neonatal age. On the other hand, the large majority of dental, maxilla-facial and ENT surgical procedures are performed in patients with a body weight ≥2 kg, beyond the neonatal age.

## 5. Conclusions

The application of uniform and shared protocols aims to improve the management of pediatric and neonatal patients with, on the one hand, the possibility of reducing SSIs and, on the other hand, containing the phenomenon of antimicrobial resistance, with the consequent rationalization of resources and costs. Our panel of experts thinks that, in the face of extremely heterogeneous prescriptions in real life characterized by the excessive and often inappropriate use of antibiotics in SAP, our document represents a balanced and shared text, derived from an extensive discussion, which can be extraordinarily beneficial for patients and, more generally, for the health system.

Due to there being a lack of pediatric data for the majority of dental, maxillo-facial and ENT surgeries and the fact that the recommendations for adults are currently used indicates the need for ad hoc studies to be rapidly planned for the most deficient areas. This seems even more urgent for interventions such as those involving the first airways since differences in the composition of the respiratory microbiota in children compared to adults implies the possibility that SAP schemes ideal for adults may not be equally effective in children. There is, however, once again the problem of identifying pediatric subjects as special subjects, and not as small adults, for whom the use of antibiotics should be carefully evaluated according to the specific characteristics of the various stages of development. Specific studies with new diagnostic methods on the respiratory microbiota in patients of different age ranges undergoing dental, maxillo-facial and ENT surgical procedures are needed.

When our consensus document is implemented by Italian Scientific Societies, it will be interesting to analyze its clinical and economic impact in our geographical context. However, our recommendations could be generalized also to low- and middle-income countries, where the impacts of simple, cost-effective, sustainable and adaptable strategies on the reduction in morbidity risk and the associated costs have recently been highlighted.

## Figures and Tables

**Table 1:** Main maxillo-facial and ear-nose-throat (ENT) surgery procedures, divided into clean or clean/contaminated and/or frankly contaminated.

Clean Procedures	Clean/Contaminated and/or Frankly Contaminated Procedures
Cleft lip and cleft palate repair Insertion of tympanostomy tubes * Tympanoplasty * Stapedectomy * Cochlear implant placement Septoplasty Thyroidectomy Parathyroidectomy Neck dissection Salivary gland surgeries Removal of lymphangiomas Excision of lateral and medial neck cysts and fistulas Tonsillectomy Adenoidectomy	Insertion of tympanostomy tubes ** Tympanoplasty ** Stapedectomy ** Rhinosinus endoscopic surgery Oral cavity resection Laryngectomy Pharyngectomy Tracheotomy Removal of upper airways tumor masses

* In subjects without infection of the ear canal and/or middle ear; ** in subjects with chronic infectious middle ear disease with or without cholesteatoma or involving drainage from an infected site.

**Table 2:** Surgical antimicrobial prophylaxis (SAP) for neonates and children undergoing dental, maxillo-facial or ear-nose-throat (ENT) surgeries.

Clinical Scenario	Recommendation
Dental surgery	No peri-operative antibiotic prophylaxis is recommended. Oral amoxicillin or ampicillin ev (50 mg/kg for both) should be administered during the 30 min before surgery if the operation involves the manipulation of gum tissue or the periapical region of the teeth or involves the perforation of the oral mucosa and the subject has already suffered from endocarditis, has already been operated with the application of prosthetic material, has a cyanogenic congenital heart disease which has not yet fully repaired, has a congenital heart disease already repaired with the application of prosthetic material in the 6 months following surgery or has been transplanted and has developed valvulopathy. No prophylaxis is recommended in subjects with prosthetic implants.
Maxillo-facial fracture surgery	Pre-operative antibiotic prophylaxis with oral amoxicillin 50 mg/kg is recommended within 30 min prior to surgery when the surgery involves the mandible. Prophylaxis is not recommended in the case of maxillary or zygomatic surgery.
Temporo-mandibular surgery	Pre-operative antibiotic prophylaxis with cefazolin in a single dose of 30 mg/kg (maximum dose 2 g) EV is recommended in the 30 min before surgery.
Cleft lip or cleft palate repair	Peri-operative prophylaxis with ampicillin/sulbactam at a dose of 50 mg/kg (as ampicillin) EV is recommended to be administered within 30 min before surgery.
Ear surgery	Peri-operative antibiotic prophylaxis is not recommended for clean surgery, whereas it is recommended in cases of clean/contaminated or contaminated operation and for cochlear implant placement. When antibiotic prophylaxis is indicated, it is recommended to administer cefazolin as a single dose of 30 mg/kg (maximum dose 2 g) EV within 30 min before surgery.
Endoscopic paranasal cavity surgery and septoplasty	It is recommended to administer peri-operative antibiotic prophylaxis with cefazolin 30 mg/kg (maximum dose 2 g) EV within the 30 min before surgery. No antibiotic prophylaxis is recommended in septoplasty.
Clean head and neck surgery	No perioperative antibiotic prophylaxis is recommended in the case of neonatal or pediatric patients undergoing clean head and neck surgery (i.e., thyroidectomy, parathyroidectomy, salivary gland surgeries, the removal of lymphangiomas and the excision of lateral and medial neck cysts and fistulas).
Clean-contaminated head and neck surgery	In the case of a neonatal or pediatric patient undergoing clean-contaminated ENT surgery (i.e., oral cavity resection, laryngectomy, pharyngectomy, tracheotomy or maxillary of upper airways tumor masses), peri-operative antibiotic prophylaxis with cefazolin 30 mg/kg (maximum dose 2 g) EV administered within 30 min before surgery combined with metronidazole 15 mg/kg (max 500 mg) is recommended.
tonsillectomy, adenoidectomy or both	No antibiotic prophylaxis is recommended.

## Data Availability

All the data are included in the manuscript.
